# Use of Spatial Information and Search Strategies in a Water Maze Analog in *Drosophila melanogaster*

**DOI:** 10.1371/journal.pone.0015231

**Published:** 2010-12-03

**Authors:** Julien Foucaud, James G. Burns, Frederic Mery

**Affiliations:** 1 Laboratoire Evolution, Génomes et Spéciation, UPR-CNRS 9034, Gif/Yvette, France; 2 Department of Biology, University of Toronto at Mississauga, Mississauga, Canada; University of Missouri, United States of America

## Abstract

Learning the spatial organization of the environment is crucial to fitness in most animal species. Understanding proximate and ultimate factors underpinning spatial memory is thus a major goal in the study of animal behavior. Despite considerable interest in various aspects of its behavior and biology, the model species *Drosophila melanogaster* lacks a standardized apparatus to investigate spatial learning and memory. We propose here a novel apparatus, the heat maze, conceptually based on the Morris water maze used in rodents. Using the heat maze, we demonstrate that *D. melanogaster* flies are able to use either proximal or distal visual cues to increase their performance in navigating to a safe zone. We also show that flies are actively using the orientation of distal visual cues when relevant in targeting the safe zone, i.e., *Drosophila* display spatial learning. Parameter-based classification of search strategies demonstrated the progressive use of spatially precise search strategies during learning. We discuss the opportunity to unravel the mechanistic and evolutionary bases of spatial learning in *Drosophila* using the heat maze.

## Introduction

Most organisms live in environments in which resources, mates, competitors and parasites are heterogeneously distributed. The ability to acquire, select, and retain relevant information about the environment may thus strongly affect their fitness [1], [2] and influence the evolution of animal cognition [3]. Spatial learning and memory have been intensively studied in humans [4], [5] and model species such as mice [6], [7] and rats [8], [9]. Such research has generated insights on what genes (e.g., the *NMDAR1* gene in rodents; [10]), brain regions (e.g., the mammalian hippocampus; [11] but see [12]), and eco-evolutionary factors (e.g., habitat range, [13]; competition, [14]) are involved in shaping spatial learning abilities.

Among the various protocols used in the laboratory to study spatial learning, the Morris water maze and its variants [15], [16] are undoubtedly the most popular. In the original version of the task, a rodent is challenged to find a hidden escape platform in an open water tank. After repeated trials, wild type rodents usually succeed in using distal visual cues to orient themselves and quickly reach the escape platform. The water maze enables measurement of spatial learning ability, as well as an evaluation of the strategies animals use to locate the platform [17], [18]. Its success can be attributed to its reliability, simplicity and the ease of manipulating extra-maze and intra-maze cues [16].

In insects, spatial learning has been predominantly studied in bees and ants in relation to their striking homing and navigational skills [19], [20]. In contrast, *Drosophila melanogaster* spatial learning ability has received limited attention despite its status as a model species for the study of learning and neurobiology [21]–[23]. To date, only three experimental protocols have been developed to explore spatial learning in *D. melanogaster*. First, the ‘flight simulator’ apparatus allows the study of operant learning of safe and unsafe flight directions based on their association with visual cues [24]. This complex but customizable setup has promoted the understanding of decision making [25], [26] and visual pattern recognition mechanisms [27]–[29]. Second, the ‘heat box’ apparatus is used to study the spatial operant learning of a safe and unsafe zone within a dark corridor using tactile and/or ideothetic cues [30]–[32]. While less amenable to spatial cue manipulations than the flight simulator, the heat box apparatus' fast and robust procedure has enabled high-throughput testing of the thermal reinforcement properties [33], [34] and neural pathways involved in this learning task [35]–[37]. Third, the detour paradigm of Neuser et al. [38] has been essential in uncovering the molecular and cellular basis of orientation memory in *D. melanogaster*. These apparatus have provided important insights into the neurobiology of spatial learning but do not allow the study of search strategies. However, the development of such strategies may reveal how spatial maps are constructed and thus represent a key parameter in the study of the evolution of cognitive functions.

Spatial learning studies in *Drosophila* are still hindered by the lack of a behavioral apparatus that would enable free-moving flies to learn about a large, potentially complex, two-dimensional space where spatial cues can be manipulated and where potential search strategies can be analyzed. We present here a novel operant spatial learning apparatus conceptually based on the Morris water maze, but using high temperature as a negative reinforcer. Complex versions of this type of maze, using heated and cooled water flows, have been used successfully in cockroaches [39] and crickets [40]. Our electric version of the experimental apparatus, hereafter called ‘heat maze’, is a simple, small, and cost-effective device. We used this apparatus to demonstrate that *D. melanogaster* wild type flies are able to precisely locate a safe zone using various types of visual cues. Most interestingly, *Drosophila* flies can rely on the information provided by distal visual cues to improve performance over repeated trials, hence displaying spatial learning. We also show that *D. melanogaster* display a progressive use of spatially precise search strategies to locate the safe zone when provided reliable distal visual cues.

## Results

**Figure 1:**
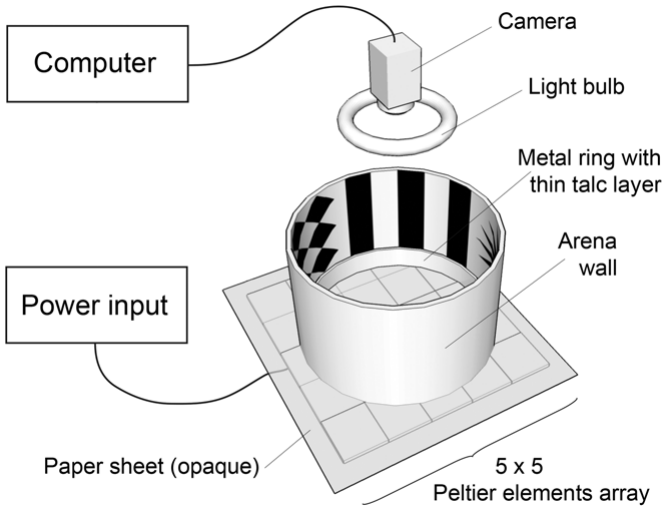
Schematic representation of the heat maze apparatus. Note: The floor of the maze consists of a 5×5 array of Peltier elements, below an opaque sheet of paper, that provides the maze with both negative reinforcement and one safe zone (analogous to the platform of the Morris water maze). Ground landmarks (in non-spatial experiments) or wall patterns (in spatial experiments) provide visual cues. The paths of flies were video-tracked using a USB camera and computer software.

Our goal was to design a behavioral test apparatus similar to the Morris water maze, adapted for insects in general and *D. melanogaster* in particular (Figure 1). We used heat as a negative reinforcer for three main reasons: the ecological relevance of this stimulus [41], the previous successful use of heat as a reinforcer in the heat box learning paradigm [31], and the ability to precisely control this parameter. Previous attempts have been made in other insect species using a metal arena floor coupled to a water heating and cooling system [40], [42]. Our choice was rather to use small electric devices, Peltier elements, whose surface temperature can be controlled.

**Figure 2:**
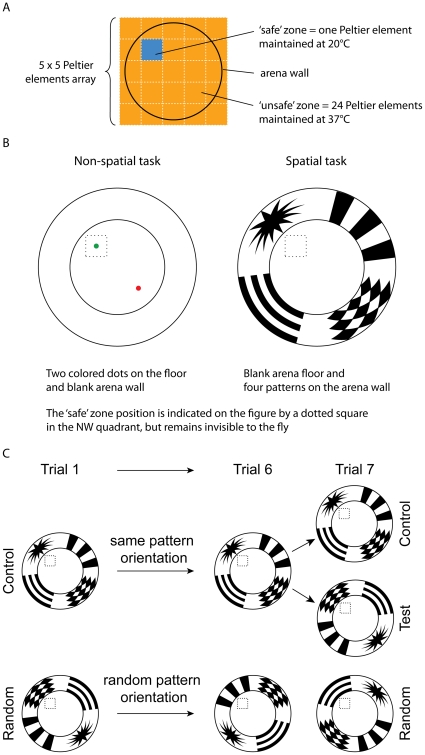
Experimental apparatus and setups. Note: (A) Heat reinforcement of the maze. (B) Proximal and distal cues setup of experiment 1. (C) Training procedures of control, test and random flies during experiment 2.

Our heat maze thus consists of a large circular arena (18 cm in diameter) on top of a 5×5 array of Peltier elements (individual dimensions : 4×4 cm), heated to aversive levels (37°C) everywhere except at one “safe” zone (20°C; see Materials and Methods section for details; Figure 2A). The size of our safe zone (4×4 cm) represents 6.28% of the floor surface, approximately 7 times and 21 times the relative size of the Morris water maze platforms used for mice and rats, respectively [16]. Arena wall consisted of a 20 cm high blank paper sheet surrounding a 5 cm high metal ring covered with talc to prevent any escape. Cues were positioned on the floor or walls of the arena depending on the experimental procedure (Figure 2). We video-tracked the position of an individual fly with clipped wings and its locomotion during consecutive five or ten minute trials.

We conducted two different experiments to test for non-spatial and spatial learning by *D. melanogaster* in the heat maze.

### Experiment 1: Response to proximal and distal visual cues

We first investigated the ability of male and female *D. melanogaster* to precisely locate a safe zone within the heat maze after repeated trials in the presence of either proximal or distal visual cues (Figure 2B).

For proximal cues, the wall of the maze was blank, and one green dot (Ø = 0.8 cm) was placed on the floor in the centre of the safe zone and one red dot (Ø = 0.8 cm) was placed in its symmetrical position in the opposite quadrant (i.e. in the unsafe zone; Figure 2B). The proximal visual cues on the safe zone acts as beacons, thus the fly can learn to discriminate between the two differently colored dots to get to the safe zone efficiently. Twenty flies (n_males_ = 10 and n_females_ = 10) went individually through three trials of ten minutes each. For this and every subsequent experiments, the starting position of individual flies was randomized within the three quadrants not enclosing the safe zone at each trial, and inter-trial intervals lasted 10 seconds when flies were handled approximately 10 cm above the floor surface (but still within arena wall) with a paintbrush.

For distal cues, the floor of the maze remained blank, but the wall was covered with four patterns: vertical stripes, diamonds, horizontal stripes and a 16-branches star (Figure 1 & 2B). All patterns were placed in the centre of a quadrant and measured 50° vertically and horizontally. Stripes were 12° wide as seen from the centre of the arena, similar to the size used in the flight simulator apparatus [43]. In preliminary experiments using no heat reinforcement, flies showed a slight preference for the stripe patterns. We thus alternatively used either the star or diamonds patterns as the cue in the quadrant of the safe zone during our experiments. Eighty flies (n_males_ = 40 and n_females_ = 40) went individually through three trials of ten minutes each, with an inter-trial interval of ten seconds. The distal visual cues do not afford the opportunity to use discrimination learning.

**Figure 3:**
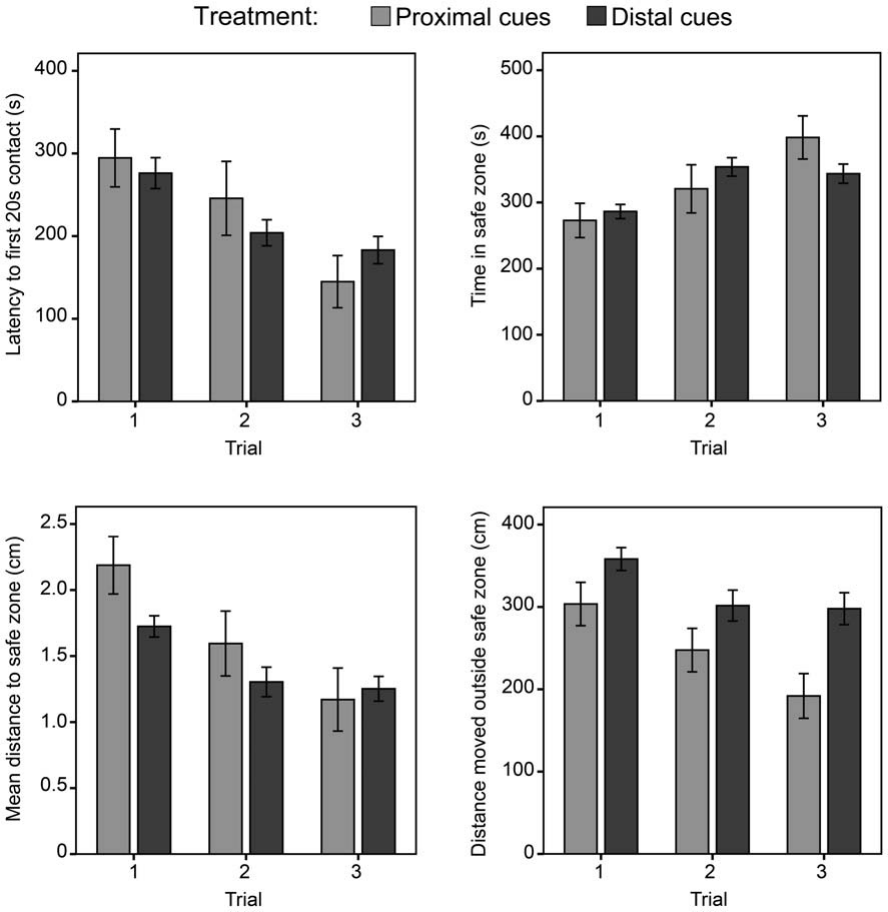
Performance of flies in locating the safe zone using either proximal cues (i.e., non-spatial task; n = 20) or distal visual cues (i.e., spatial task; n = 20). Note: Flies presented either the non-spatial and spatial tasks improved their performance in locating the safe zone: the latency before a 20 s contact with the safe zone decreased (proximal: *F*_(1,52)_ = 11.57, *p* = 0.001; distal: *F*_(1,224)_ = 16.32, *p*<0.001), the time spent in the safe zone increased (proximal: *F*_(1,52)_ = 16.03, *p*<0.001; distal: *F*_(1,223)_ = 10.67, *p* = 0.001), the mean distance to the safe zone decreased (proximal: *F*_(1,52)_ = 10.23, *p* = 0.002; distal: *F*_(1,224)_ = 13.81, *p*<0.001) and the distance moved outside the safe zone declined (proximal: *F*_(1,52)_ = 9.33, *p* = 0.003; distal: *F*_(1,224)_ = 8.37, *p* = 0.004) with trials. The comparison between non-spatial and spatial tasks revealed no significant differences (latency before a 20 s contact: *F*_(1,294)_ = 0.17, *p* = 0.68; time in safe zone: *F*_(1,293)_ = 0.01, *p* = 0.93; mean distance to the safe zone: *F*_(1,294)_ = 3.18, *p* = 0.08), except for a larger distance travelled in the spatial task (*F*_(1,294)_ = 12.96, *p*<0.001). Male and female flies were pooled. Error bars indicate standard errors.

As shown in Figure 3, flies improved their overall performance in locating the safe zone throughout trials with reliable proximal or distal visual cues. This trend was highly significant for all measured statistics (Figure 3). After the third trial, most flies remained within the safe zone well above chance level in both the proximal and distal cues configurations (>40-fold chance level; Figure S1).

When comparing the relative performance of flies subjected to proximal vs. distal cues, we found no significant difference for most variables (Figure 3). However, flies travelled significantly more distance when in presence of distal visual cues than in presence of proximal cues (Figure 3). It is noticeable that, in presence of distal cues, flies seemed close to asymptotic performance after three trials; this is not the case for flies in the presence of proximal cues. Additional training might thus uncover asymptotic performance differences between the two types of information.

We did not detect any gender difference in the increase of performance to locate the safe zone using proximal cues (latency before a 20 s contact with the safe zone: *F*_(1,52)_ = 0.85, *p* = 0.36; time spent in the safe zone: *F*_(1,52)_ = 0.60, *p* = 0.44; mean distance to the safe zone: *F*_(1,52)_ = 0.70, *p* = 0.41; distance moved outside the safe zone: *F*_(1,52)_ = 2.51, *p* = 0.12; Figure S2). However, when presenting distal cues, we observed a pronounced gender effect for global activity within the maze, males travelling much longer distance than females for every trial (*F*_(1,232)_ = 50.82, *p*<0.001; Figure S3). While the two first trials did not lead to significant differences between genders in other measures of performance, males usually showed a poorer performance than females in the third trial (latency before a 20 s contact: t_(78)_ = 3.07, *p* = 0.003; time in safe zone: t_(78)_ = −2.06, *p* = 0.043; but mean distance to the safe zone: t_(78)_ = 1.65, *p* = 0.103).

### Experiment 2: Spatial Learning and search strategies

In this experiment, we attempted to disentangle how flies were using spatial information provided by distal visual cues to improve their performance in locating the safe zone (i.e. did the flies use the patterns on the maze wall to learn the position of the safe zone? What search strategies did flies use?). Using a blank floor and the previously described wall patterns, three groups of 40 female flies each went individually through seven trials of five minutes, with an intertrial time of ten seconds. For the ‘control’ group, the position of the patterns relative to the safe zone remained constant throughout the seven trials (Figure 2C). For the ‘test’ group, the position of the patterns relative to the safe zone was constant for the six first trials (i.e. a training phase), but was rotated 180° on the seventh trial (i.e. a test phase). For the ‘random’ group, flies were given unreliable wall patterns by randomly rotating the arena wall between each of the seven trials.

**Figure 4:**
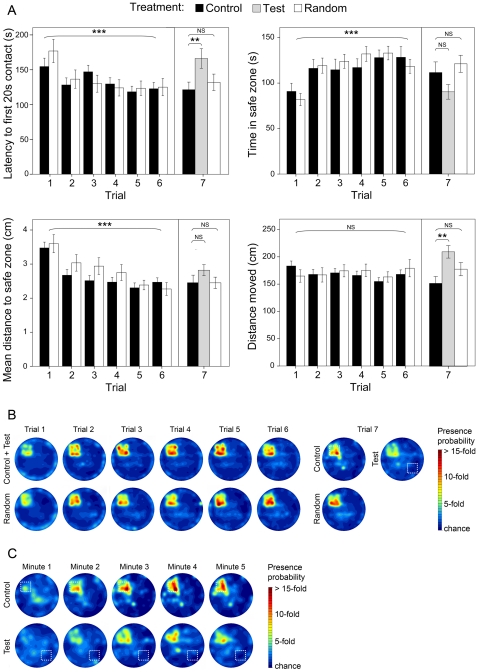
Spatial learning in *Drosophila* flies. Note: (A) Latency before 20 s contact, time spent in the safe zone and mean distance to the safe zone showed a significant improvement after six 5 minute trials for both control and random groups of flies. Most measured variables showed this trend: the latency before a 20 s contact diminished linearly (linear within subject contrast: *F*_(1,77)_ = 19.6, *p*<0.001), the time spent in the safe zone increased (*F*_(1,79)_ = 20.6, *p*<0.001), the mean distance to the safe zone declined (*F*_(1,78)_ = 24.84, *p*<0.001). In contrast, the distance moved outside the safe zone did not improve (*F*_(1,78)_ = 3.8, *p* = 0.06). Wall pattern reversal significantly altered the ability for test flies to locate the safe zone as shown by their latency to reach the safe zone and their overall distance moved (Dunnett's test for multiple comparison: latency fo first 20 s contact: *p* = 0.004; distance moved: *p* = 0.002). Test flies were also undistinguishable from naïve flies for three variables (latency before 20 s contact: t_(115)_ = 0.01, *p* = 0.99; time in safe zone: t_(112)_ = −0.75, *p* = 0.45; distance moved outside safe zone: t_(115)_ = −1.67, *p* = 0.10). Control flies were significantly different from naïve flies for all variables (latency before 20 s contact: t_(117)_ = 2.17, *p* = 0.03; time in safe zone: t_(112)_ = −2.36, *p* = 0.02; distance to safe zone: t_(117)_ = 3.51, *p* = 0.001; distance moved outside safe zone: t_(117)_ = 2.02, *p* = 0.05). Performance from the random group of flies improved throughout trials (linear within subject contrast: latency before 20 s contact: *F*_(1,38)_ = 11.6, *p* = 0.002; time in safe zone: *F*_(1,39)_ = 9.89, *p* = 0.003; distance to safe zone: *F*_(1,39)_ = 10.3, *p*<0.001; distance moved outside safe zone: *F*_(1,39)_ = 0.3, *p* = 0.92). Statistical significance is indicated with asterisks for linear within subject contrasts and Dunnett's tests (***: p<0.001; **: p<0.01; NS: non-significant). N = 120 female flies. (B) Presence probability plots of control, test and random groups during each 5 minute trials of experiment 2. All groups developed a positional preference for the safe zone through trials. During the seventh trial, test flies displayed a reduced positional preference for the safe zone in comparison to control flies. The white dashed square represents the expected position of the safe zone relative to distal visual cues. (C) Presence probability plots of control and test flies during each minute of the seventh trial of experiment 2. Wall pattern reversal resulted in a both delayed and less intense positional preference for the safe zone in test flies when compared to control flies.

Flies displayed a significant improvement of navigation towards the safe zone over the six trials when patterns reliably predicted the position of the safe zone, as shown by three out of four variables (Figure 4). Test flies, which experienced a reversal of the relative position between the safe zone and wall patterns, performed poorly during the seventh trial in locating the safe zone in comparison to control flies. Indeed, test flies took significantly longer before staying within the safe zone for 20 s and covered significantly more distance outside the safe zone than control flies (Figure 4). The performance of test flies was not significantly different from that of naive flies (i.e., flies during their first trial) for three of the four variables (Figure 4). This result contrasted with control flies who were significantly different from naive flies for all variables (Figure 4). A close examination of the presence probability plots of control and test flies during that seventh trial showed that test flies were generally slower at getting to the safe zone and never reached presence levels similar to control flies (Figure 4C).

Interestingly, flies of the random group, in which the patterns did not reliably indicate the position of the safe zone, also improved their performance over trials and performed even as well as control flies throughout the course of the experiment (Figure 4). Again, during the test phase, performances of random and control flies were indistinguishable (Figure 4).

**Figure 5:**
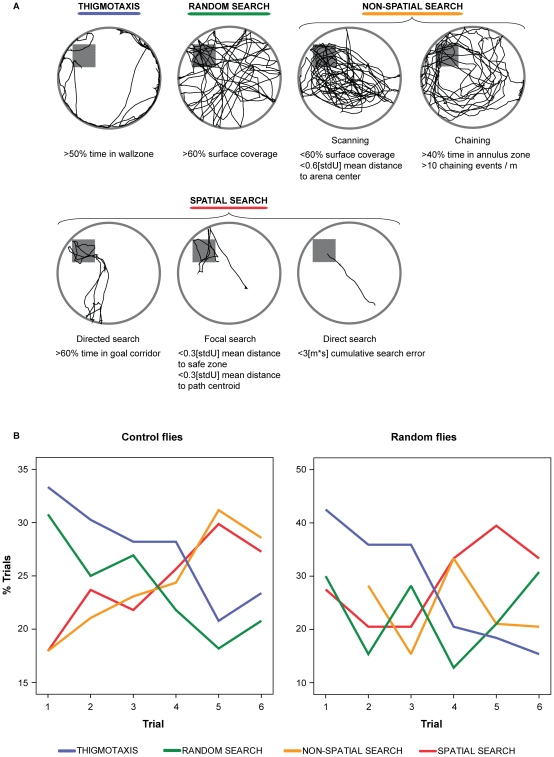
Parameter-based classification of search strategies. Note: (A) Detailed presentation of the algorithm-based classification of search strategies, including their key parameters and cut-off values. Exclusion of different strategies was performed in a particular order (from the most to the least spatial) because of the less specific nature of certain strategies. [stdU] represents radius length. (B) Control flies significantly decreased their use of thigmotaxis and random search in favor of non-spatial and spatial search strategies. In contrast, random flies failed to show a significant trend toward using a particular strategy, despite abandoning thigmotaxis. Our algorithm successfully classified more than 96% of the trials performed. N = 120 female flies.

Parameter-based classification of search patterns yielded additional insights about the nature of control and random flies' performance (see Materials and Methods for details). Though both groups of flies improved their performance in locating the safe zone during the training phase, only control flies displayed a significant tendency to progressively use more spatially precise search strategies (Figure 5B). Indeed, control flies gradually abandoned the use of thigmotaxis and random search strategies in favor of non-spatial and spatial strategies in the course of the training phase (multinomial general lineal model: Wald Chi-Square = 5.1, *p* = 0.02). On the contrary, random flies tended to progressively avoid thigmotaxis, but failed to progressively use spatially precise search strategies (multinomial general lineal model: Wald Chi-Square  = 3.005, *p* = 0.08).

## Discussion

Our work demonstrates that free-walking *D. melanogaster* are able to use various types of visual cues to improve their performance when locating a preferred area and can develop search strategies adapted to the environmental conditions.

Our first experiment shows that *D. melanogaster* flies improve their performance in avoiding a negatively reinforced area after repeated trials in environments providing reliable proximal or distal visual cues. Both types of cues led to a rather similar performance in locating the safe zone, indicating no *a priori* reliance of a single type of cue in normal *Drosophila* orientation. This was expected given current knowledge in both insects and mammals. From mammalian studies using the Morris water maze, spatial information from proximal and distal landmarks seem to be integrated [44], [45] and navigation could rely on egocentric (i.e., related to self) or allocentric strategies (i.e., related to external environment) depending on the availability and reliability of cues together with goal distance [46]. In insects, numerous field studies have underlined the simultaneous use of ego- and allocentric strategies for homing in Hymenoptera [47]–[49]. Further experiments are needed to explore the potential simultaneous use of distal and proximal visual cues and of their integration on the behavioral level in *D. melanogaster*. This experiment also suggests a slight gender difference in locating the safe zone, but only when distal cues were present. In this case, male individuals were more active, but not as fast as females in locating the safe zone. In mammalian studies, spatial learning is often sexually dimorphic with males outperforming females [50], [51] (but see [52]). This finding is generally related to the particular sexual selection pressures and range size of focus species [13], [53], [54]. In *Drosophila melanogaster*, some authors have argued a sex-biased dispersal in favor of females from natural populations [55], [56] (but see [57]), that would comply with our observations of better female spatial learning. An alternative hypothesis is that *Drosophila* males and females could differ in their visual acuity, e.g. [58], [59], explaining the poorer use of distal cues by males.

Most importantly, our second experiment demonstrates that free-walking flies actively use distal cues to navigate in an open field. Trained flies that experienced a reversal of the wall patterns displayed a poor performance similar to naïve flies, in sharp contrast with control trained flies. The reversal of wall patterns, by triggering a “resetting” of the flies' behavior, revealed the flies' use of spatial information gathered during previous trials. Moreover, our results show that flies increased their use of spatial search strategies when provided reliable distal visual cues. Overall, these experiments demonstrate the occurrence of spatial learning in our trained *Drosophila* flies. The neural mechanisms underlying such a performance in the heat maze remain to be elucidated. Previous studies of other visual learning tasks (such as the flight simulator and the detour paradigm) revealed that the main center for visual input processing in relation to locomotion, orientation and landmark recognition in *Drosophila* seems to lie within the central complex [28], [29], [38], [43], [60]. Alternatively, the mushroom bodies may mediate place memory using distal visual cues, as inferred for *Periplaneta* cockroaches [39], [42]. At the behavioral level, we show that the increase of performance in the spatial task occurred via a progressive switch from poor search strategies (thigmotaxis, random search) to more accurate strategies (non-spatial and spatial search strategies). This progress in the use of spatially precise search strategies indicates that wild type flies are able to acquire a spatial representation of their environment, based on the integration of distal visual cues.

Interestingly, flies that were trained using a random relative position of wall patterns also performed significantly better with experience. Contrary to flies trained using a reliable spatial information, random flies did not increase their use of spatial search strategies. However, these flies did learn the only spatial information possible in this setup, by decreasing their natural tendency for thigmotaxis. At least, two hypotheses could explain the improvement of random flies in our setup. First, random flies were able to extract a reliable visual cue from our apparatus despite a strict control over 85% of the total inner surface of the maze, and used that cue to orientate. This effect has already been advocated in a previous study of spatial memory in crickets using a similar maze when no reliable cue indicated the correct position of the safe zone [40]. In our apparatus, only cues visible through the open top (i.e. comprised between 20 and 60 cm) were left uncontrolled. However, our analysis of the random flies search strategy failed to display an emergence of spatial strategies in the course of the training phase, which argues against this hypothesis. Second, because distal visual cues were not reliable, random flies may have learned to pay more attention to other aspects of the test. For instance, some individuals may have been more likely to learn that the safe zone was a certain distance from the wall and thus utilize “chaining”. We however did not find such tendency in the search strategy analysis. Taken all together, our results suggest that *Drosophila* may develop an optimal search strategy in accordance with the stability of the available environmental cues.

Our work demonstrates that spatial learning in *Drosophila* can be precisely unraveled using the heat maze. As such, it illustrates the power of the simple conceptual approach of the Morris water maze [15]. However, our apparatus differs slightly from the original paradigm. In its present version (manually-controlled power input and temperature), we could not technically perform a true ‘probe’ trial with the heat maze (i.e. a final trial without platform to test for learning; [16]) as the time required to change and re-stabilize the temperature of a Peltier element was too long. We are currently developing a version of the heat maze with computer-controlled temperature for every Peltier element of the apparatus that will enable shortening the inter-trial interval time to a few seconds, allowing for probe trials in future studies.

We argue that the heat maze apparatus could be used for the study of spatial learning with both mechanistic and evolutionary perspectives. The heat maze permits a thorough exploration of genetic, developmental and environmental factors influencing spatial learning and search strategies in *Drosophila*. We expect this to be a fruitful methodological approach given the opportunity to couple it with the large libraries of *Drosophila* mutants or the powerful GAL/UAS system, which span genes and brain regions that have been proven to play crucial roles in learning and memory in other tasks (e.g., the *rutabaga* or *dunce* mutants, the central complex and mushroom body regions; [43], [61], [62]). Besides genetic effects, a variety of environmental factors influencing spatial learning and retrieval could be explored in our apparatus by manipulating either the source of information (e.g., investigating the use of multiple potentially conflicting cues; [63]), either the experience of tested individuals (e.g., investigating aging or addiction-related effects; [64], [65]). Our standardized protocol also opens up prospects in the evolutionary study of spatial learning in *Drosophila* species. For instance, cactophilic flies species from the Sonoran desert exhibit various dispersal strategies due their strict host specialization and the different spatial distribution of their host species [66]. The putative correlation between home range size and spatial learning abilities could be tested using these species in our paradigm. Such an evolutionary approach could in turn benefit more mechanistic approaches by exposing natural variants in spatial learning and memory.

## Materials and Methods

### Animals

Adult (two to five days old) wild-type *D. melanogaster* flies (collected in Chavroches, France, and laboratory adapted over several generations) were used. To prevent flying during the course of the experiments, flies were anaesthetized on ice and their wings were clipped 12 to 24 hours before introduction to the heat maze. All individuals were maintained in small groups (less than 20 individuals) on a standard yeast-cornmeal agar food under a 12L:12D cycle at room temperature.

### Apparatus: the heat maze

We built an 18 cm-diameter arena, limited by a 5 cm high metal ring covered by a thin talc layer to prevent the flies from escaping the arena (see Figure 1). Surrounding this metal ring, a circular 20 cm high wall made of blank white paper was used to display (or not) distal visual cues (see below).

To allow a simple design and satisfactory control of the arena floor temperature, we chose to use an array of 25 Peltier elements (arranged in a 5×5 grid; each one measuring 4×4 cm; Figure 1 & S1A). The surface temperature of each Peltier element is directly related to the amount of electrical power applied and can be finely tuned manually or via computer-control, assuring a precision below 1°C. For 24 Peltier elements, the temperature was set to 37°C, as this has proved to be a non-lethal negative reinforcement in previous experiments in this and other learning paradigms (e.g., [35]). We used the remaining Peltier element as a safe zone—an equivalent to the platform in the Morris water maze—and set its surface temperature to a permissive 20°C. The 18 cm diameter arena thus consisted of a large ‘unsafe’ 37°C zone and a ‘safe’ 20°C platform of 4×4 cm (Figure 1). On top of this array of Peltier elements, we laid a regular sheet of paper (80 g/m^2^) as a floor surface. This paper surface allowed us to set up a very steep temperature gradient between safe and unsafe zone, to render safe and unsafe zones visually and texturally indistinguishable, and to remove potential odor cues simply by replacing the floor surface. The whole heat maze apparatus was placed in a 60×60×60 cm wooden box to control for more distal visual cues. Air temperature within the box was 25°C±1°C.

To record an individual fly's position, we placed a commercial web camera together with a circular light bulb on top of the apparatus (Figure 1). We video-tracked the flies' position using Ethovision XT 7 (Noldus, Wageningen, Netherlands).

### Data analysis

The main statistics computed were: time spent in safe zone, distance moved outside safe zone, mean distance to safe zone, and latency to spend 20 consecutive seconds within the safe zone. For the latter statistic, we chose to use a 20 s contact period as a measure of the fly's decision to remain in the safe zone because the first contact with the safe zone were usually simple crossings and did not appear to reflect a decision. This simple difference with water maze experiments makes it more difficult to interpret results pertaining to decisions and search strategies (i.e. when has the fly learnt the safe position?). Unfortunately, no objective event similar to the reaching of the escape platform in the Morris water maze could be drawn from the heat maze apparatus. Our decision was to consider that flies had “attained” the safe zone when they remained on it for 20 consecutive seconds, because this amount of time appears clearly out of the distribution of times spent on any random location on the heat maze, thus reflecting a behavioral shift. Repeated measure analysis of variance and *t* tests were used to establish significant differences between trials (by fitting simple linear contrast) and treatments using SPSS v17. Presence probability plots were constructed using the MASS package [67] of the R statistical software [68].

To investigate the use of spatial search strategies during the course of place learning in *Drosophila*, we classified searching behaviors of the flies using a parameter-based algorithm [18], [69]. Similarly to rodents [17], we could discriminate between seven search strategies used in the course of learning (Figure 5A). “Thigmotaxis” refers to the strong tendency to remain in close proximity of the arena wall. It may indicate some form of anxiety. Thigmotaxis is usually followed by a “random search” strategy where individual flies explore the entire arena surface. We thus detected this strategy using a high threshold for surface coverage. Some flies display non-spatial strategies including “scanning”, in which exploration is mainly confined to the central area of the maze, where visual cues are more salient. We detected this strategy principally using a small threshold on the mean distance to the center of the arena (Figure 5). Another non-spatial strategy is “chaining”, in which exploration is mainly performed in an annulus zone at the correct distance between the safe zone and the arena wall. We used both a criteria of a minimum presence in the annulus zone (a 3 cm wide zone centered on the middle of the radius of the arena) and the occurrence of at least 10 chaining events per meter of distance travelled. A chaining event was scored when the fly serially visited three of the 12 virtual goals evenly placed at the annulus distance from the arena wall. Otherwise, flies can display spatial strategies. These include the “directed search” strategy, in which exploration is mainly confined to the corridor between the starting point and the safe zone. We detected this strategy by computing the proportion of time spent in a 6 cm corridor between the starting point and the center of the safe zone for every trial. The “focal search” strategy is characterized when a fly perform its search in the immediate neighborhood of the safe zone. In this case, both the mean distance to zone and the mean distance to the centroid of the travelled path must be very short (i.e., less than 3 cm). Finally, the best possible strategy is “direct search”, in which flies navigate straight to the safe zone. We detected this strategy using the cumulative search error variable, in which deviation from the optimal direct search is computed taking into account both the individual's starting point and mean speed during the analyzed trial. The additional parameters required for accurate classification were computed using the public software Wintrack [18] (Figure 5A). All 720 trials of experiment 2 were classified by serially excluding the respective strategies from the most to the less precisely defined (i.e., direct search, focal search, directed search, chaining, scanning, random search, thigmotaxis; [17]). Our parameter-based algorithm was able to classify more than 96% of trials. Progressive switch from loss of thigmotaxis, random search to non-spatial and spatial strategy was analyzed by fitting a multinomial general lineal model including trials as covariate.

## Supporting Information

Figure S1 Presence probability plots of the trained flies of experiment 1 during each of the three first minutes of the three trials, using either (A) proximal cues or (B) distal cues. Note: The safe zone is located in the NW quadrant. Flies showed an increased positional preference for the safe zone through time within trials and, most importantly, through trials.
(TIF)

Figure S2 Performance of male and female flies in the non-spatial task of experiment 1. Note: Latency before the first 20 consecutive seconds contact, time spent in the safe zone, mean distance to the safe zone and distance moved outside the safe zone all showed no difference in performance level between genders during three 10 minutes trials in the non-spatial version of the task.
(TIF)

Figure S3 Performance of male and female flies in the spatial task of experiment 1. Note: Male and female *D. melanogaster* usually show no difference in performance during the first two trials, but do so for their latency before the first 20 consecutive seconds contact and time spent in the safe zone during the third trial. Gender significatively influenced the distance moved outside the safe zone through the experiment in the non-spatial version of the task.
(TIF)
