# Supplementary figures and images for: Use of Spatial Information and Search Strategies in a Water Maze Analog in Drosophila melanogaster
Source: PLoS One. 2010 Dec 3;5(12):e15231. doi: 10.1371/journal.pone.0015231 (PMC2997081; doi:10.1371/journal.pone.0015231)

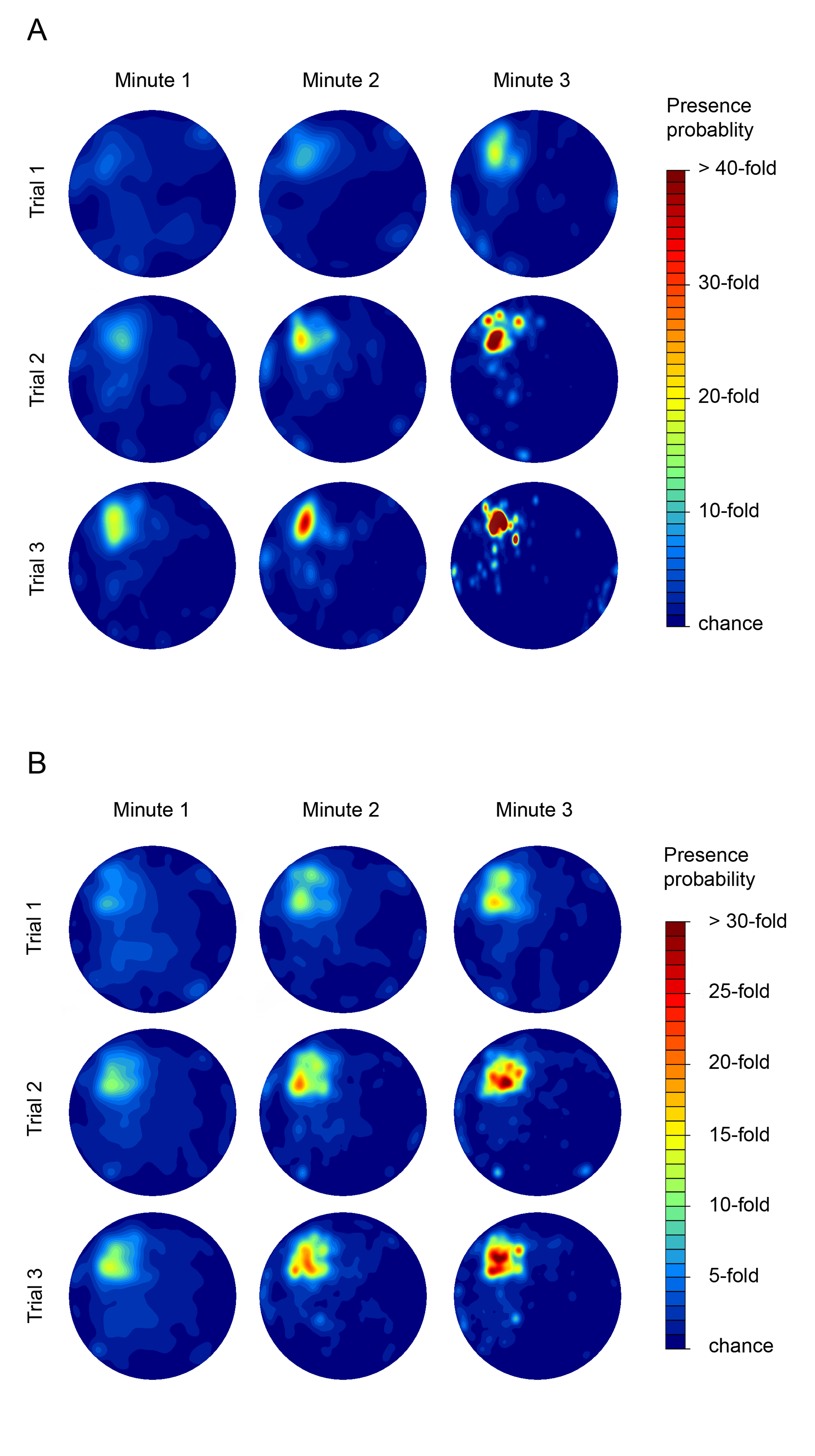

Supplement: Figure S1 — Presence probability plots of the trained flies of experiment 1 during each of the three first minutes of the three trials, using either (A) proximal cues or (B) distal cues. Note: The safe zone is located in the NW quadrant. Flies showed an increased positional preference for the safe zone through time within trials and, most importantly, through trials. (TIF) [file pone.0015231.s001.tif]

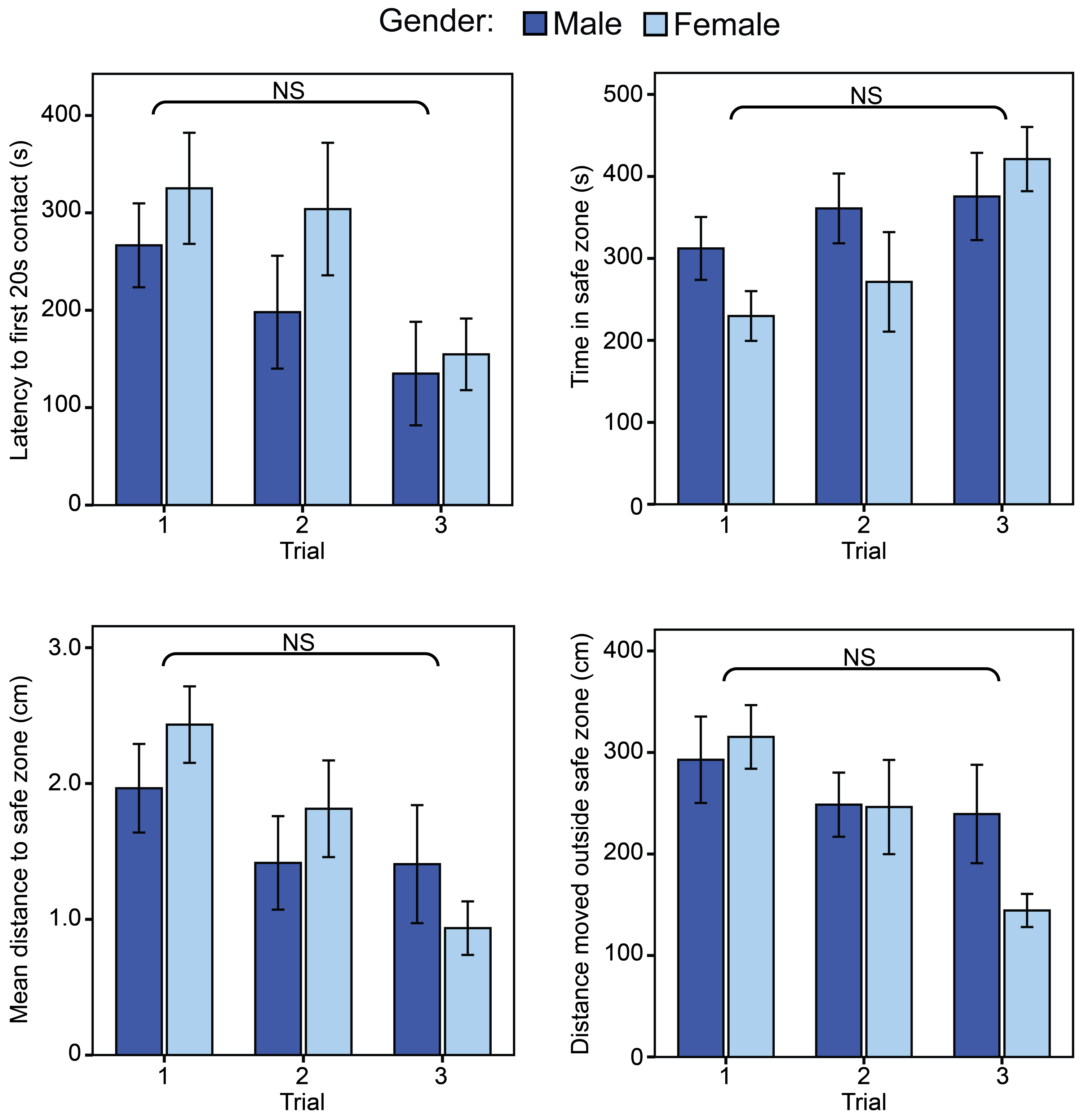

Supplement: Figure S2 — Performance of male and female flies in the non-spatial task of experiment 1. Note: Latency before the first 20 consecutive seconds contact, time spent in the safe zone, mean distance to the safe zone and distance moved outside the safe zone all showed no difference in performance level between genders during three 10 minutes trials in the non-spatial version of the task. (TIF) [file pone.0015231.s002.tif]

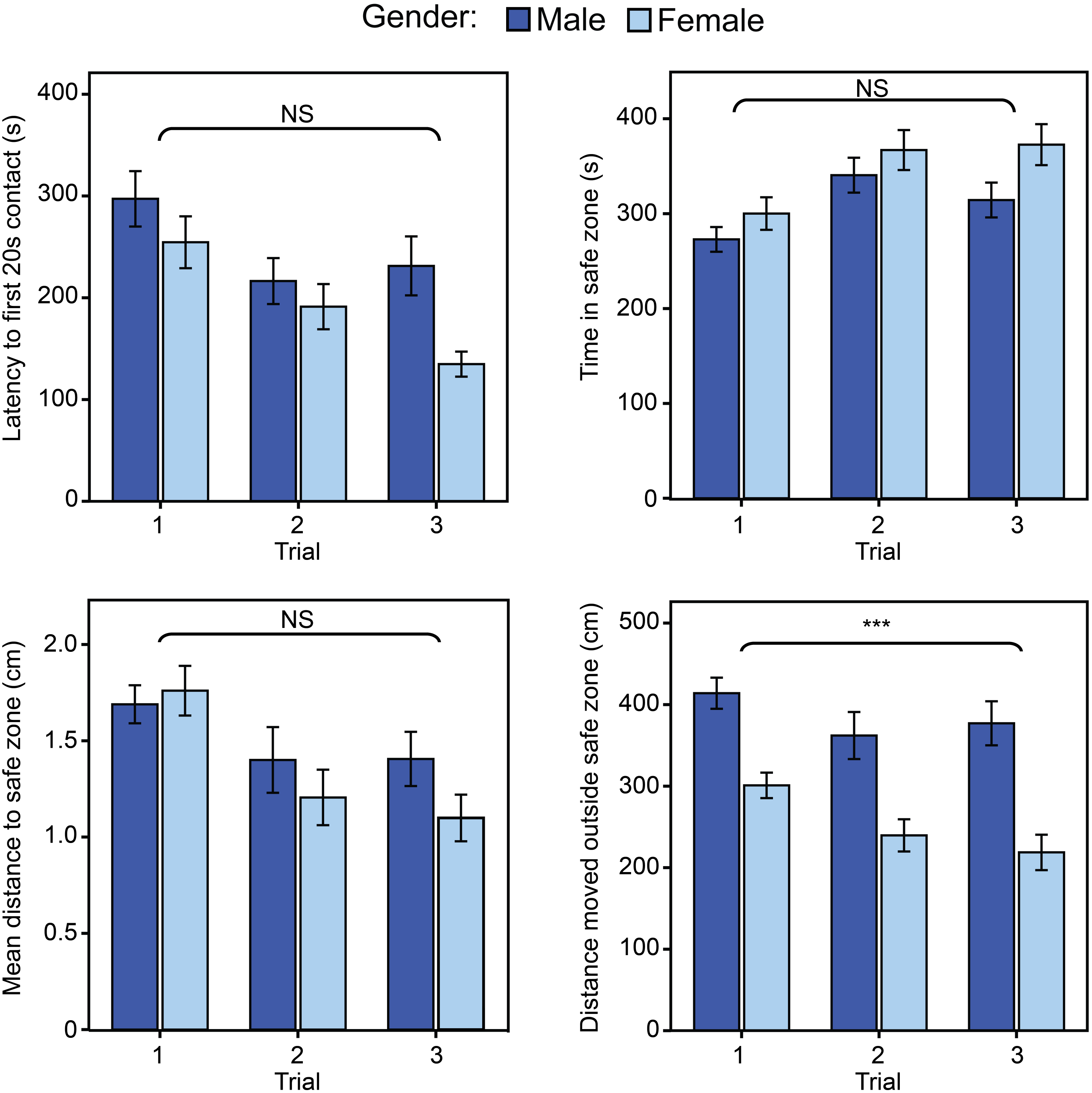

Supplement: Figure S3 — Performance of male and female flies in the spatial task of experiment 1. Note: Male and female D. melanogaster usually show no difference in performance during the first two trials, but do so for their latency before the first 20 consecutive seconds contact and time spent in the safe zone during the third trial. Gender significatively influenced the distance moved outside the safe zone through the experiment in the non-spatial version of the task. (TIF) [file pone.0015231.s003.tif]
